# The feasibility of using actigraphy to characterize sleep in Rett syndrome

**DOI:** 10.1186/s11689-018-9227-z

**Published:** 2018-02-27

**Authors:** Alyssa M. Merbler, Breanne J. Byiers, John J. Garcia, Timothy J. Feyma, Frank J. Symons

**Affiliations:** 1 Department of Education Psychology, University of Minnesota, 250 Education Sciences, 56 E River Rd, Minneapolis, MN 55455 USA; 2 Sleep Health, Gillette Children’s Specialty Healthcare, 200 University Ave E, Saint Paul, MN 55101 USA; 3 Pediatric Neurology, Gillette Children’s Specialty Healthcare, 200 University Ave E, Saint Paul, MN 55101 USA

**Keywords:** Rett syndrome, MECP2, Sleep, Actigraphy

## Abstract

**Background:**

Rett syndrome (RTT) is a neurodevelopmental disorder primarily caused by mutations in the MECP2 gene. Sleep problems are reported by the majority of caregivers of individuals with RTT.

**Methods:**

The present study aimed to replicate and extend previous work about the feasibility of measuring sleep with an actigraph device in a sample of girls with clinically diagnosed RTT (*N* = 13, mean age = 9 years, 5 months). Participants wore an actigraph device day and night for seven consecutive days. Materials also included a parent-completed sleep diary to measure bedtime, duration of nighttime sleep, and daytime sleep, and the Child Sleep Habit’s Questionnaire (CSHQ).

**Results:**

The means for the sample as measured by actigraphy were 492.3 min (SD = 47.3) of total night sleep (TNS), 76.0% (SD = 6.7) sleep efficiency, 86.0 min (SD = 34.2) of wake after sleep onset, and 46.1 min (50.8) of sleep when parents reported a nap occurring. Parents reported 589.7 min (SD = 53.6) of TNS, 15.9 min (SD = 12.0) of WASO, and 93.6 min (SD = 66.8) of daytime sleep according to sleep diaries, with all parents reporting at least one nap during the week. Relations were found between sleep characteristics and seizure status and CSHQ total scores. No age-related changes were observed for any sleep characteristic, regardless of collection method. Five of nine participants above the cutoff score on the CSHQ indicate the need for further evaluation for a sleep disorder.

**Conclusions:**

Overall, actigraphy was feasible in this community-based sample of girls with RTT. The results replicated some aspects of previous studies of sleep in RTT (e.g., no age-related changes in total nighttime sleep or efficiency). Some participants met the American Academy of Sleep Medicine guidelines for recommended total sleep time, with others showing too much or too little sleep. Each of the three methods for describing sleep presented its own advantages and challenges. Future work should be prospectively designed, validate the use of actigraphy in this population, and include a typically developing comparison sample to improve the precision of our understanding of sleep in RTT.

## Background

Rett syndrome (RTT) is a rare neurodevelopmental disorder characterized by myriad debilitating health complications and numerous developmental and behavioral problems [1, 2]. RTT affects approximately one in 10,000–15,000 live female births (it is exceedingly rare in males) and is most often caused by mutations in the methyl-CpG-binding protein 2 gene (MECP2) [3]. Most females with RTT (i.e., “classic RTT”) follow a pattern of seemingly typical development from birth until 6–18 months, after which they experience a significant regressive period. The regression involves a loss of acquired skills including purposeful hand movement and language abilities [2]. There are numerous health issues including difficult to manage seizures and autonomic dysfunction marked by cardiac arrhythmias, breathing abnormalities, and peripheral skin temperature anomalies [1, 4].

Considering the numerous health and behavioral issues, it is not surprising that sleep problems are commonly reported in RTT. The presence of an abnormal sleep pattern is a supporting characteristic included in the diagnostic criteria for classic and atypical RTT [5], and 79–85% of caregivers reported their children experienced at least one sleep problem, such as frequent nighttime waking, spells of screaming or laughing at night, or daytime sleepiness [6–8]. Clinical and preclinical studies have examined melatonin and circadian activity rhythm cycles in RTT. Results indicated abnormal melatonin cycles in a sample of two girls with RTT [9] and abnormal circadian activity rhythms in mice with RTT compared to wild type controls [10, 11].

Understanding the prevalence and presentation of sleep problems in RTT has important clinical implications. A qualitative investigation by McDougall, Kerr, and Espie [12] describes that caregivers of individuals with RTT report their child’s sleep dysfunction affects their sleep and energy levels, marriage, mood, and social life, as well as their child’s mood and energy [12]. In other populations, indices of sleep dysfunction, such as sleepiness, sleep quality, and sleep duration, are correlated with a child’s school performance and daytime functioning [13], and behavioral regulation and neurobehavioral function [14]. Documenting the prevalence and severity of sleep dysfunction is a critical step towards the development of future clinical trials targeting control of circadian rhythms and sleep/wake cycles among individuals with RTT. Further, abnormal sleep patterns may reflect underlying physiological dysfunction and may therefore serve as a non-invasive marker that could be used to evaluate the effects of pharmacological and/or environmental manipulations designed to improve physiological outcomes in this population. To date, two clinical trials of therapeutics targeting core symptoms of RTT included parent-reported sleep questionnaires as outcome measures, including the Children’s Sleep Habit Questionnaire (CSHQ). The CSHQ is a questionnaire designed to screen children to identify those who need further evaluation for a pediatric sleep disorder [15] and has been used to characterize sleep in other developmental disability populations, such as autism spectrum disorders (ASD) [16], Angelman syndrome [17], and Williams syndrome [18]. While it is being used as an outcome measure in a clinical trial for individuals with RTT, there is currently no published literature on its use in this population, and thus, further investigation is needed.

Investigations into sleep dysfunction in RTT clinical samples have yielded inconsistent results. Some studies have documented increased total sleep time compared to typically developing norms when grouped by age [7], while others have found less total sleep time (TST; nighttime + daytime sleep) [19] or less total nighttime sleep (TNS) with increased daytime sleep compared to comparison samples [20, 21]. Ellaway et al. [7] found that younger girls with RTT slept less than developmental norms, but older teens with RTT slept more. Frequent night wakings, increased sleep onset latencies, and low sleep efficiency have also been reported [19, 21]. Preliminary evidence suggests that differences in total daytime sleep may be related to seizure status, as Ellaway and colleagues also found that girls with RTT who had a seizure disorder had more daytime sleep and more of their total sleep time was during the day than those without a seizure disorder [7]. Polysomnography (PSG) studies have had conflicting results, with some indicating relatively normal sleep architecture [22, 23], while others reported increases [23] or decreases [24] in duration of rapid eye movement (REM) sleep.

There are many potential explanations for the divergent findings across the studies conducted to date. It should be noted that many of the studies were completed prior to the seminal work by Amir and colleagues [3] implicating MECP2 in the development of RTT. Therefore, it is plausible that some of the participants in the earlier studies had distinct, but clinically similar syndromes. In addition, the studies vary considerably with respect to sample sizes, recruitment methods, and data collection methods. Data collection methods have included caregiver questionnaires [6, 8, 25], direct observations [20, 21], caregiver-completed sleep diaries [7], PSG [22–24], and actigraphy [19]. Each of these methods is associated with its own set of advantages and disadvantages. Survey data are based on subjective caregiver reporting that may require parents to recall sleep behaviors, sometimes over long time scales. This method has the advantage of including the caregivers’ full, long-term knowledge of their children and their sleep patterns, but may lead to over-reporting of memorable events due to psychological biases, making it unclear whether overall results reflect the child’s current sleep patterns. Issues can also arise due to differences in interpretation (e.g., what is considered a sleep problem by a parent), which may differ across caregivers or between caregivers, researchers, and clinicians [12]. Direct observational methods allow in-home recording of sleep behaviors, as well as more sensitivity to analyze a participant’s typical sleeping pattern, but may not be sensitive to brief awakenings that occur, as behavior is not recorded continuously but intermittently across intervals throughout the night [20, 21]. Finally, PSG offers insight into the specific stages of sleep and its cortical physiology, but often requires participants to spend the night in a medical setting attached to equipment, removing the participant from their home environment and usual bedtime routine. The across-study differences and limited replication within collection methods may contribute to the apparent inconsistencies.

One approach that preserves the “home environment” for recording while allowing continuous recording is actigraphy, a method of data collection increasingly applied in sleep research because of its sensitivity and ability to record in-home for extended periods of time [26]. When worn day and night, a watch-like actigraph transduces movement which can be used to make inferences about sleep/wake periods. Actigraphy has been used to characterize sleep in typically developing populations as well as populations with developmental disabilities [26], including individuals with motor impairments associated with neurodevelopmental disorders such as cerebral palsy [27] and Angelman syndrome [28].

In the only published study of using actigraphy to measure sleep in RTT, McArthur and Budden [19] characterized the sleep patterns of nine girls before and during a melatonin treatment trial. Sleep was measured with actigraphy as well as with caregiver-completed sleep diaries for seven consecutive nights before treatment began and for 10 weeks during treatment. Although specific inclusion/exclusion criteria were not described specific to presence or absence of sleep disorders, all participants were enrolled in a sleep intervention trial. Given that parents are unlikely to consent to a treatment trial in the absence of a clinical problem, it is likely that enrolled participants had pre-existing sleep problems. For this reason, it is likely that the results may not reflect the average sleep patterns among a non-referred sample of individuals with RTT. Therefore, additional studies using actigraphy to examine sleep patterns among a broader sample of individuals with RTT are warranted.

The purpose of the present study was to demonstrate the feasibility of using actigraphy to characterize sleep in a sample of girls with clinically diagnosed RTT. To do so, we partially replicated the baseline phase of McArthur and Budden’s clinical sleep trial in which they used actigraphy as part of their outcome measures. In the current sample, girls were not participating in a sleep treatment trial. Further, we present data from three different measures of sleep to characterize sleep in the sample: actigraphy, parent report via sleep diary, and the Child’s Sleep Habits Questionnaire. It was hypothesized that actigraphy would be feasible and that our sample would have sleep characteristics that deviated from age-specific developmental norms. We were also able to explore several RTT relevant clinical variables, including parent-reported seizure status.

## Methods

### Participants and setting

Following IRB approval, participants were consecutively recruited through a local parent support network in the Midwest. Thirteen Caucasian girls with clinically diagnosed RTT syndrome and confirmed MECP2 mutations (mean age = 9 years, 5 months, range = 1 year, 8 months––17 years, 1 month) and their families participated in the study. The sample included 11 girls who met criteria for classic RTT, one who met criteria for atypical RTT, and one with a MECP2-related disorder. The participant with the MECP2-related disorder had a clinical diagnosis of atypical RTT, but did not meet diagnostic criteria based on a review of her medical records. Twelve of 13 participants had confirmed MECP2 mutations. All families that completed the consent process completed the collection protocol. See Tables 1 for full participant characteristics.

**Table 1:** Participant characteristics

ID	Age	Diagnosis	Mutation	Seizure status	Gross motor function	Feeding	Medications^+^			
							Sleep	Seizure	Pain	Spasticity
1	1 year, 8 months	Atypical	p.P152R	None	Walks with assistance	Eats finger foods	N	Y	Y	N
2	4 years, 3 months	Classic	p.P362fs	None	Walks with assistance	Fed by mouth	N	N	N	N
3	6 years, 7 months	Classic	p.E137_L386del	Suspected	Walks with assistance	Fed by mouth	N	Y	N	N
4	8 years, 2 months	Classic	p.R168X	None	Walks with assistance	Fed by mouth	N	N	Y	Y
5	8 years, 3 months	Classic	p.R168X	Controlled	Cannot sit unsupported	Fed by mouth	N	Y	N	N
6	8 years, 3 months	Classic	p.P152R	Uncontrolled	Cannot sit unsupported	Fed by mouth, has g tube	N	Y	N	N
7	8 years, 10 months	Classic	p.R255X	Controlled	Cannot sit unsupported	Has g tube	N	N	Y	N
8	9 years, 4 months	MECP2-related disorder	p.R106W	Controlled	Walks independently	Eats with utensils with some spilling	N	N	N	N
9	10 years, 4 months	Classic	p.A131fs	Uncontrolled	Walks with assistance	Fed by mouth	N	Y	N	N
10	11 years, 9 months	Classic	Exon 4 deletion	Uncontrolled	Cannot sit unsupported	Eats finger food, has g tube	Y	Y	N	Y
11	12 years, 7 months	Classic	Deletion between exon 3 and 4	Uncontrolled	Cannot sit unsupported	Fed by mouth	N	Y	N	N
12	15 years, 4 months	Classic	p.P272fs	Controlled	Cannot sit unsupported	Fed by mouth, has g tube	N	Y	N	N
13	17 years, 1 month	Classic	p.K144X	Uncontrolled	Walks with assistance	Eats finger food	Y	Y	N	N

^+^Several participants were also on other medications for health conditions, such as acid reflux and constipation

### Data collection

After IRB approval and informed consent was obtained, all caregivers were mailed a package containing a Philips Actiwatch 2 (Philips Respironics, Bend, Oregon) and a sleep diary to record their child’s sleep. The actigraph was programmed to collect the data in 30 s epochs day and night for seven consecutive days. Caregivers were instructed to place the device snuggly on their child’s wrist. The watch was place on the ankle for participant 1, due to recommendations for children under 2 years old [29]. Although hand stereotypy is present in many of those with RTT, it does not occur during sleep [30, 31]. Thus, the watch was placed on the wrist consistent with other actigraphy studies, including McBudden and Arthur [19].

#### Questionnaires

Ad hoc questionnaires (described below) were included with the sleep watch to gather more information about each participant’s overall and daily health and mood. This included items related to each participant’s alertness, additional medications taken, pain experienced, and seizure activity for each day of the collection period. Due to the addition of new questionnaires during the study period, not all families completed all questions (completion rates described below).

The CSHQ is a parent-completed questionnaire aimed at gathering information about different dimensions of children’s sleep [15]. The questionnaire includes items about sleep onset and bedtime behavior, sleep duration, morning and night wakings, sleep anxiety, behavior during sleep, daytime sleepiness, parasomnias, and breathing of school-aged children. Items are scored on a 3-point scale based on how often they occurred in the previous week (1 = rarely or 0–1 times, 2 = sometimes or 2–4 times, and 3 = usually or 5–7 times), and higher scores indicate more sleep-related problems. Of the 45 items on the questionnaire, 33 are scored for a score range of 33–99, and a score of 41 or more indicating a need for further evaluation of a potential sleep disorder. Eleven of 13 families received the CHSQ (participants 2 and 3 did not due to changes in study protocol). We evaluated internal consistency of the questionnaire using Cronbach’s alpha.

A sleep diary is a tool that caregivers complete daily in the home environment to indicate the time their child was put to bed, the time their child fell asleep, any night wakings, and the time their child woke up in the morning, as well as any daytime sleep. Sleep diary tools are often included in actigraphy studies for verification of times and identifying artifact [7, 29]. Sleep diaries were completed by parents for each day of actigraphy recording and were used to verify actigraph data during the editing process. Twelve of 13 families completed the sleep diary for a total of 78 of 91 nights (85.7%). Participant 9 did not return the sleep diary, and thus, daytime sleep and parent-reported TNS, and total sleep time (TST) could not be calculated.

### Analysis

**Fig. 1:**
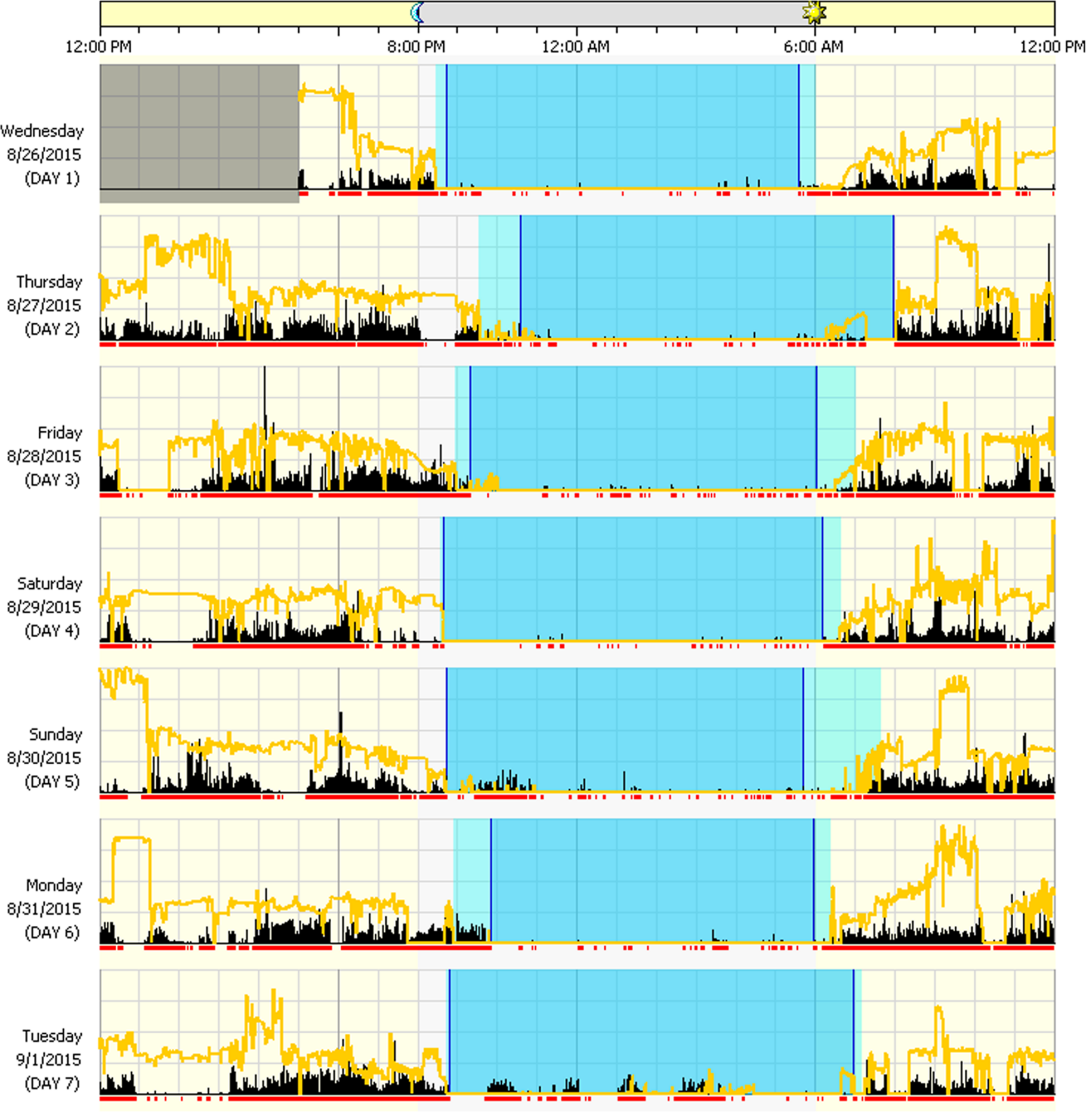
Example actigraphy report. Example actigraphy report (participant 5). Sleep patterns are displayed for individual days. The vertical black bars and the red line under each day indicate movement, and the yellow line indicates light (natural or artificial). Lighter shaded intervals are rest, and darker shaded intervals are sleep

Actigraphy data were analyzed using Phillips Actiwatch 6 software. Settings included wake threshold set at low sensitivity and sleep interval detection set to 20 epochs (10 min) for sleep onset and sleep end. We chose low sensitivity as it has been demonstrated that this brand of actigraph better aligns with PSG when on the “low” setting [32]. The actigraphy software generates graphs of movement and ambient light data over time for each participant (see Fig. 1 for an example). The graphs were inspected visually and edited by two independent coders. Bedtime was set by identifying the point in time at which lights were turned off (as identified by a sudden decrease in ambient light) that was closest in time to the parent-reported bedtime from the sleep diary. The interobserver agreement coefficients for TNS, sleep efficiency, and WASO were in the acceptable range. Onset of the rest interval (related to sleep onset latency), however, had somewhat lower total agreement (i.e., agreements divided by agreements plus disagreements) of 76.4%, when a 10-min difference between raters was used as the cutoff for disagreements. The mean discrepancy between raters for rest interval onset was 9.8 min (SD = 17.3), with a median disagreement of 2.0 min.

For each participant, the following characteristics were calculated using the software and averaged across all days of the collection: total nighttime sleep (TNS), sleep efficiency, sleep onset latency (onset latency), daytime sleep, and wake time after sleep onset. Parent reported TNS and daytime sleep were calculated from completed sleep diaries. Due to insensitivity of the actigraphy analysis to detect brief periods of sleep, naps were only counted if the parent-reported nap duration was at least 10 min (Table 2).

**Table 2:** Sleep variable definitions

Characteristic	Definition
Total nighttime sleep (TNS)	Total sleep between sleep onset and offset once a person was in bed
Total sleep time (TST)	The sum of total night sleep and daytime sleep
Sleep onset latency (onset latency)	The time it takes to fall asleep from lights out
Sleep efficiency	The percent of time in an interval that the person is sleeping, calculated as [time asleep/(total interval time––invalid time) × 100]
Wake after sleep onset (WASO)	The amount of time a person spends awake after they initially fall asleep
Total daytime sleep	The amount of time spent asleep when parents report a nap of at least 10 min

Means and standard deviations of sleep characteristics measured by actigraphy and sleep diaries were calculated for each participant. The data were not normally distributed, so we used non-parametric analyses to examine relations between variables. Spearman correlations were used for relations between sleep variables (e.g., TNS), age, and CSHQ total scores, and Mann-Whitney *U* tests were used to examine sleep variables by seizure status. Additionally, an effect size was calculated for seizure status and sleep efficiency from the results of the Mann-Whitney *U* test [33]. Because of the preliminary nature of the study, type I errors (i.e., finding possibly spurious relationships) were judged to be less problematic than type II errors (i.e., missing possible relationships), and the nominal type I error rate was set at 0.1.

To determine whether the participants in the sample met the guidelines for age-appropriate sleep duration and for sleep quality, each participant’s results were evaluated against previously published guidelines. Average total sleep time (TST; TNS plus daytime sleep) values (based on both actigraphy-derived and parent-reported sleep data) were evaluated against the American Academy of Sleep Medicine (AASM) guidelines for recommended total sleep time by age group, which are endorsed by the American Academy of Pediatrics and aligned closely with the National Sleep Foundations guidelines [34, 35]. Average sleep efficiency and onset latency values for each participant were compared to cutoff values used by Souders et al. [16] from the Children’s Hospital of Philadelphia Sleep Center. Based on this work, sleep efficiency values lower than 85% and onset latency values greater than 30 min indicate potential problems with sleep quality.

## Results

### Sleep characteristics from actigraphy

Actigraphy data were successfully collected for all 13 participants. There was a 7.7% loss (seven nights) of data across the full sample due to non-compliance. Non-compliance included issues such as removing the watch for water activities or bathing and failing to put it back on until the following morning. During follow-up conversations after the data collection, all parents reported the collection week went smoothly overall, and there were no reported issues with the actigraph device or the process of collecting actigraphy data. Additionally, there was no reported discomfort or injury due to the device.

An example actigraphy report is presented in Fig. 1, and average values for sleep statistics are presented in Table 3. The mean TNS for the sample as measured by actigraphy was 492.3 min (SD = 47.3). For the 12 participants with complete data, 4 (33.3%) fell within the recommended range of TST, 6 (50%) fell below the recommended range, and 1 (0.08%) was above the recommended range when using actigraphy-derived values. Twelve of 13 participants (92%) had a mean sleep efficiency value below 85%, and 10 of 13 participants (76.9%) had a mean onset latency within the appropriate range (< 30 min).

**Table 3:** Average sleep statistics (SD) by participant based on actigraphy (act), sleep diary (parent), and the CSHQ

ID	TNS (min/SD)		WASO (min/SD)		Daytime sleep (min/SD)		Actigraphy-only Variables		TST status*	CSHQ score
	Act.	Parent	Act.	Parent	Act.	Parent	Efficiency (%/SD)	Latency (min/SD)	Act/parent	
1^a^	463.6 (74.9)	469.3 (199.7)	127.8 (45.0)	21.4 (37.6)	4.7 (11.4)	12.5 (18.2)	72.4 (12.2)	29.6 (29.1)	Too little/too little	39
2	443.1 (53.5)	615.0 (48.2)	117.4 (25.9)	4.3 (11.3)	27.4 (24.8)	117.5 (55.7)	71.2 (4.4)	22.1 (20.8)	Too little/appropriate	NA^c^
3	521.8 (134.5)	660.7 (134.5)	83.8 (39.3)	19.3 (51.0)	132.4 (95.4)	196.3 (63.5)	73.6 (21.4)	30.7 (40.0)	Appropriate/too much	NA^c^
4	539.5 (32.2)	600.0 (32.4)	36.9 (12.0)	7.5 (15.0)	0 (0)	11.25 (14.4)	84.1 (4.8)	21.9 (15.4)	Appropriate/appropriate	42^+^
5	450.1 (51.0)	540.0 (52.7)	96.3 (62.9)	17.1 (45.4)	88.7 (34.9)	139.3 (57.3)	74.5 (10.2)	23.3 (25.7)	Appropriate/appropriate	54^+^
6	416.3 (107.9)	585.0 (72.8)	116.9 (27.6)	10.0 (15.5)	17.6 (26.8)	127.5 (101.2)	69.0 (14.9)	24.4 (37.3)	Too little/appropriate	NA^b^
7	566.1 (52.3)	620.0 (43.1)	24.5 (7.4)	22.5 (29.5)	52.1 (71.1)	87.0 (64.0)	87.8 (8.6)	19.5 (38.3)	Appropriate/appropriate	42^+^
8	502.6 (37.6)	540.0 (47.4)	62.8 (23.0)	10.7 (18.8)	0 (0)	5.0 (11.2)	81.8 (3.0)	18.6 (16.9)	Too little/appropriate	34
9^d^	479.5 (115.3)	–	92.2 (57.1)	–	–	–	71.1 (17.0)	24.3 (19.1)	–	38
10	472.6 (59.6)	597.5 (39.6)	127.3 (31.7)	0.5 (1.2)	22.8 (25.4)	100.0 (29.5)	70.3 (7.6)	16.6 (17.8)	Too little/appropriate	43^+^
11	461.5 (151.4)	600.0 (64.8)	109.1 (34.8)	40.7 (66.4)	58.0 (64.4)	162.9 (93.0)	68.2 (17.0)	75.1 (83.2)	Too little/too much	45^+^
12	564.4 (77.1)	662.1 (28.0)	55.6 (35.9)	4.9 (12.0)	144.1 (20)	146.7 (13.7)	81.7 (8.2)	61.4 (66.7)	Too much/too much	NA^b^
13	519.2 (46.1)	587.1 (41.9)	67.6 (31.3)	32.1 (32.9)	5.8 (14.3)	17.5 (27.5)	83.1 (6.6)	19.9 (16.4)	Appropriate/too much	38
Group means	492.3 (47.3)	589.7 (53.6)	86.0 (34.2)	15.9 (12.0)	46.1 (50.8)	93.6 (66.8)	76.0 (6.7)	29.8 (17.7)		37.3 (2.2)

^*^Compared to the American Academy of Sleep Medicine’s total sleep time recommendations for the appropriate age bracket [34]

^+^At or above CSHQ cutoff score [15]

^a^Actigraphy was placed on the ankle due to age

^b^Score not calculated due to excessive missing data

^c^Questionnaire not completed

^d^Sleep diary not completed

### Parent-reported sleep behaviors

Sleep diaries were successfully collected for 12 of 13 participants with a 12.1% loss (11 nights) of data due to non-compliance among completed forms. Parents did not report any complications or problems using the diary in follow-up conversations. One parent requested additional instructions for completing the sleep diary, including an example of a completed sleep diary. Some parents, however, did not distinguish between or consistently report when they put their child in bed with the lights off *and* when their child fell asleep, which made calculating parent-reported onset latency for all participants in the sample impossible. Additionally, some of the sleep diary days had ambiguous shading, creating less certainty in calculating total night sleep. For example, some parents’ sleep interval shading ended with a slanted line, with half of the line corresponding to a specific time and the other half lining up with a time roughly 15 min later.

Parents reported an average of 589.7 min (SD = 53.6) of TNS, and all parents reported that their children napped at least once during the week-long collection. In comparison with the AASM guidelines, 7 of 12 participants (58.3%) were within their TST recommended range, 4 of 12 (33.3%) were above their recommended range, and 1 participant (8.3%) fell below according to the sleep diaries (see Table 3). Finally, 6 of 11 parents (54.5%) endorsed that their daughter had spells of laughter for no reason during the night.

### CSHQ

The CSHQ was returned by all 11 families who received it, but the results from two questionnaires were excluded due to missing data (18.2%). Four of remaining nine parents (44.4%) left one to two items blank. Four of the 33 scored items were excluded because they were not applicable for most of the sample (wets the bed, talks during sleep, sleepwalks, and moves to someone else’s bed). Within the margins, parents noted issues such as “uses a diaper” for the “wets the bed at night” item (*N* = 3) and “in order to give medications” or “for position changes” in reference to “adults or siblings wake up child” (*N* = 2). The internal consistency of the items used in calculating the total score in this sample was adequate (*α* = 0.85).

The average total score was 42 (SD = 6, range = 34–54). Five of 9 participants (55.6%) scored above the CSHQ cutoff score of 41. Of the items used in scoring, the items endorsed (“sometimes” or “usually”) most frequently in this sample include the following: child seems tired (88.9%), child is sleepy or falls asleep watching TV (88.9%) and riding in the car (88.9%), child wakes once during the night (77.8%), and child is restless and moves a lot during sleep (77.8%). Four parents (36.4%) endorsed teeth grinding at night, and three parents (27.3%) endorsed that their child awoke screaming, sweating, or inconsolable during the night.

### Relations between sleep and participant characteristics

**Fig. 2:**
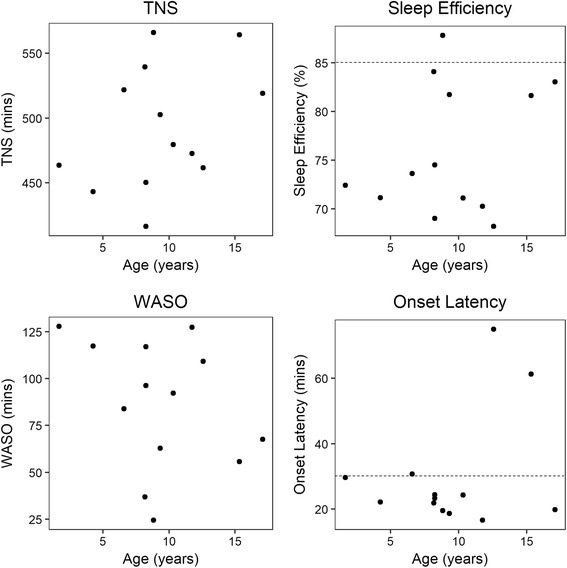
Actigraphy-derived sleep characteristics by age. Sleep characteristics obtained from actigraphy recording compared across ages (*N* = 13). Dashed lines indicate guideline for acceptable sleep efficiency (85% or higher) and onset latency (30 mins or less) from Souders et al. [16]

**Fig. 3:**
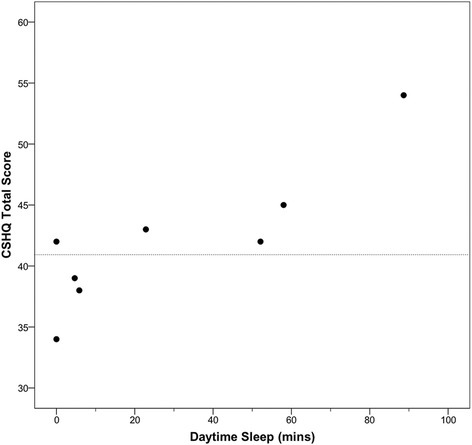
CSHQ total scores by actigraphy-derived daytime sleep. Data available for eight participants (*r* = 0.80, *p* = 0.017). Dashed line indicates CSHQ cutoff score of 41 [15]

**Fig. 4:**
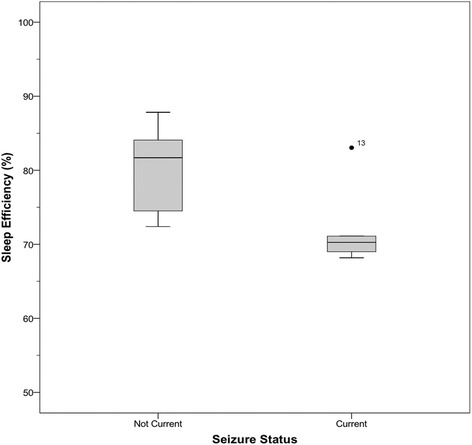
Sleep efficiency by seizure status. Sleep efficiency by seizure status (*N* = 7 no current seizures, *N* = 5 current seizures; *U* = 5; Z = 1.95; *p* = 0.051, *r* = 0.57). Participant 3 was excluded as her physicians were unsure if she was having seizures or not at the time of the study

**Fig. 5:**
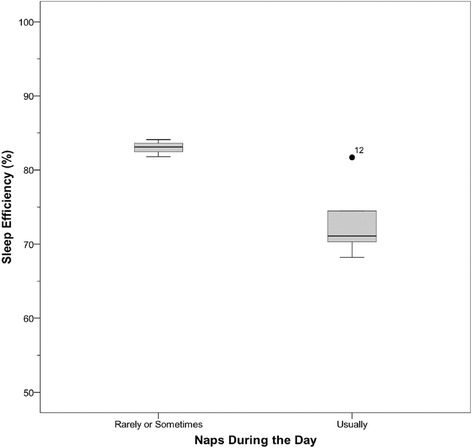
Sleep efficiency by nap frequency. Sleep efficiency grouped by nap frequency according to the CSHQ for participants 6 years or older (*N* = 4 for naps rarely or sometimes, *N* = 5 for naps often)

No age-related changes were found for actigraphy-derived TNS (*r* = 0.27, *p* = 0.38), sleep efficiency (*r* = 0.003, *p* = 0.99), onset latency (*r* = − 0.072, *p* = 0.81), or WASO (*r* = − 0.30, *p* = 0.32; Fig. 2). There were also no age-related changes for parent-reported TNS (*r* = 0.13, *p* = 0.69), WASO (*r* = 0.11, *p* = 74), or daytime sleep (*r* = 0.10, *p* = 0.76) or CSHQ total scores (*r* = − 0.09, *p* = 0.81). Total CSHQ scores were significantly correlated with actigraphy-derived daytime sleep (*r* = 0.80, *p* = 0.017, *N* = 8; Fig. 3), but no other parent-report or actigraphy-based measures. Actigraphy-derived sleep efficiency was significantly lower among those whose parents reported that they had seizures that were not controlled by medications at the time of the study (*N* = 5, *M* = 72.32%, SD = 6.10), compared to those with no history of seizures and those whose seizures were well controlled (*N* = 7, *M* = 79.05%, SD = 6.37; *U* = 5; *Z* = 1.95; *p* = 0.051, *r* = 0.57; Fig. 4). No other relations with seizure status were found. Sleep efficiency was also lower among participants age six or older who endorsed napping “usually” (*N* = 5) compared to those who napped “rarely” or “sometimes” (*N* = 4; Fig. 5). Participant 1 was excluded as she is under age six, when frequent napping is still common [36].

## Discussion

The purpose of this study was to extend the current literature on sleep patterns among individuals with RTT by demonstrating the feasibility of using in-home actigraphy, parent-reported sleep diaries, and a published sleep problem scale in a community sample of girls with RTT. Several summary-level points can be made. First, from a feasibility perspective, actigraphy was successfully used in-home by parents. There were no reported application problems or technical issues, despite the stereotypic hand movements associated with RTT. The night-by-night movement data in each participant’s report look comparable to data obtained from typically developing samples [37]. In comparison, there were more issues with the analysis of data from the sleep diaries and the CSHQ due to inconsistencies in completion and missing data. Second, from a normative perspective, the data show that most participants had appropriate onset latency, as derived by actigraphy. All but one participant, however, had low sleep efficiency (below 85%) [16], and several participants had TST outside of their recommended range according to both parent report and actigraphy measurement. Third, in relation to the indirectly measured RTT clinical characteristics, sleep efficiency was the only sleep variable significantly related to seizure status. Due to the number of participants taking sleep and seizure medications, it was not feasible to perform between-group analyses to examine differences in sleep characteristics by medication status. Visual analysis, however, did not show overlap of sleep characteristics between those prescribed and not prescribed sleep or seizure medications, and that pattern is consistent with McArthur and Budden [19], who did not find differences between those of and off anticonvulsant medication.

Aspects of the present findings that suggest poor sleep for some individuals in this sample (e.g., less TST than is recommended) align with the findings of literature reviews of sleep quality in individuals with and without intellectual disabilities that individuals with pain, genetic syndromes, and/or health conditions such as epilepsy, all of which have been documented in individuals with RTT [1, 38, 39] tend to have poorer sleep [40, 41]. The link between epilepsy and poor sleep may explain why individuals in this sample with uncontrolled seizures had lower sleep efficiency than individuals with controlled or no seizures, and differences in sleep variables by seizure status have been reported in other disability populations, such as in cerebral palsy and RTT [7, 42–44]. Further, there is a significant intra-individual variability across some sleep variables in this sample, including some participants with standard deviations of nearly 2 h for TNS across the week-long collection. This variability is also consistent with other sleep studies in developmental disabilities. For example, Hoffman, Sweeney, Gilliam, Apodaca, Lopez-Wagner, and Castillo [45] found a relation between sleep values and phenotypic differences in individuals with ASD, and Annaz, Hill, Ashworth, Holley, and Karmiloff-Smith [18] found a relation between medial history (e.g., the presence of cardiac problems) and CSHQ scores in children with Williams syndrome. Individual ranges and/or standard deviations are often not reported in investigations of sleep, though, and thus, the comparison here is limited. Further research may illuminate the reasons for within-group variability in sleep parameters in developmental disability populations.

There were no age-related changes across any of the measured sleep behaviors. In typically developing samples, some sleep characteristics remain stable across the lifespan, whereas others show developmental changes [46]. Sleep efficiency and onset latency tend to be stable and independent of age [46], and the pattern of these variables in the current sample, as measured by actigraphy, is consistent with those of other populations in that respect. Typical samples, however, also show a decrease in TNS across the lifespan, and daytime sleep tends to end around 6 years of age [36]. The current sample deviated from typical patterns in these sleep characteristics regardless of measurement method, as there were no age-related changes in TNS measured by parents or actigraphy, and all participants napped at least once during the study period. The lack of age-related changes replicated patterns found in some previous studies of sleep in RTT [19], but is inconsistent with Piazza [21], who found that nighttime sleep decreased and daytime sleep increased with age. Due to the small sample size in the current study, as well as the cross-sectional nature of the study design, longitudinal studies of change in sleep patterns in RTT would elucidate whether the lack of age-related changes observed in the current sample is due simply to large inter-individual differences obscuring such patterns, or a true lack of developmental change.

This study is the first to report scores on the CSHQ specifically in a sample of girls with RTT. In the current sample, over half of the participants scored above the cut score of the CSHQ, even after removing the four non-applicable items, suggesting that the majority of this sample may require evaluation of a sleep disorder. Additionally, CSHQ total scores were positively related to actigraphy-derived daytime sleep, indicating that individuals who endorsed more items related to sleep dysfunction also tended to nap more. Further, individuals who were reported to nap more in general on the CSHQ (and were 6 years old or older, as napping occurs in less than 10% of children by age six [36]), had lower sleep efficiency. This may indicate that excessive napping after an appropriate age leads to lower sleep efficiency or that poor sleep efficiency leads to increased napping. Future research should further examine this relationship to determine the direction of the relation, as this may have implications for sleep interventions related to low sleep efficiency. As described above, however, there were several non-applicable CSHQ items, such as sleep-walking and talking (all girls were non-ambulatory and non-verbal), that were discarded. Other items that were applicable (e.g., sleeps alone in own bed) did not contribute any variance when examining internal consistency. Although the CSHQ did show strong internal consistency in the sample overall, further research and a larger sample size are needed to continue evaluating the appropriateness and validity of the CSHQ in RTT.

It should be recognized that the current study was based on a convenience sample, and it is worth pointing out the limitations created by such an approach. Generalizing to the RTT population is not warranted given no random sampling, and the results are best considered specific to the sample. The small sample size also had reduced statistical power and limited the complexity of the analyses that could be conducted. It is possible that some of the non-significant relationships between variables, including the lack of age-related changes, may be attributable to reduced power. Further, there was no normative or disability-matched comparison sample, which limits the specificity of the conclusions (i.e., it is difficult to make claims that the findings are RTT-specific and/or abnormal), although we were able to make comparisons against national normative age-based databases. Future studies should be designed to expand the current sample, as well as add a comparison sample of age-matched typically developing females.

Possible limitations of actigraphy should also be taken into consideration when considering the results of the current study in the decision to use actigraphy to characterize sleep in this population. Collecting and analyzing sleep patterns via actigraphy has not been validated in RTT, and there exists only one validation study of actigraphy in a sample of children with developmental disorders, according to a review by Meltzer and colleagues [29]. Validation studies of actigraphy in pediatric samples showed that accuracy was quite good regarding the detection of sleep intervals but less accurate regarding wake time after sleep onset [29]. Additionally, one polysomnographic study did document an increased prevalence of periodic limb movements during sleep in a sample of individuals with RTT compared to healthy controls [47]. Although we acknowledge that this might have had a small effect on WASO detected by actigraphy, these types of movements are brief and would be unlikely to cause large errors in the estimation of total nighttime sleep. Parents, though, may also be unreliable at reporting WASO, as they may not be aware of times their child is awake at night due to being asleep themselves, and thus, this limitation of actigraphy may still be present if studies and/or clinicians are relying on parent report [48].

In addition to limitations of actigraphy itself, there seems to be a paucity of reporting specific details in the current published literature of studies using actigraphy including the device settings, scoring algorithm settings, such as sensitivity and requirements for determining a sleep epoch, and sleep characteristics definitions, making direct comparison across studies challenging. The current literature ranges from 1 to 20 consecutive minutes of sleep to count as sleep onset, which influences variables such as sleep efficiency and WASO. Similarly, many time-based characteristics, including onset latency and WASO, are dependent on scoring rules, rules which are established presumably by the research team yet not always reported. For example, “medium” sensitivity is the default and most frequently used sensitivity setting in pediatric sleep studies using actigraphy [29], but “low” has been shown to be more aligned with PSG for this device [32]. Because of a general absence of reporting of scoring rules and the effect different sensitivity settings have on the calculation of sleep characteristics, caution should be taken when comparing actigraphy data across studies.

## Conclusions

This study provides evidence on the feasibility of using actigraphy, an objective, in-home recording system, to characterize sleep in RTT. Overall, some participants had age-appropriate levels of TST and sleep onset within the recommended guidelines. On the other hand, the results indicate the presence of dysfunction for some sleep parameters in this RTT sample, specifically the continuance of daytime sleep across adolescence, low sleep efficiency, a lack of age-related changes in total night sleep, and clinically significant scores on the CSHQ. Future work should investigate the validity of using actigraphy to measure sleep in RTT, to establish an objective, in-home method to assess sleep in this population.

## Abbreviations

AASM: American Academy of Sleep Medicine
CSHQ: Children’s Sleep Habits Questionnaire
MECP2: Methyl-CpG-binding protein 2
RTT: Rett syndrome
TNS: Total nighttime sleep
TST: Total sleep time
WASO: Wake after sleep onset
